# The impact of tax reduction on enterprises’ financialization-A quasi-natural experiment based on the reduction of VAT rate

**DOI:** 10.1371/journal.pone.0293385

**Published:** 2023-12-21

**Authors:** Shu Duan, Yuzhong Lu, Yujia Cheng, Qian Liu

**Affiliations:** 1 School of Business, Suzhou University of Science and Technology, Jiangsu, China; 2 School of Public Economics and Administration, Shanghai University of Finance and Economics, Shanghai, China; Nanjing Audit University, CHINA

## Abstract

This study examines the influence of the reduction in value-added tax (VAT) rates in China during 2018 and 2019 on corporate financialization. By employing a difference-in-differences model and utilizing data from Chinese A-share listed companies between 2017 and 2020, we assess the effects of tax reduction policies. Moreover, it achieves this outcome through three main pathways: alleviating financing constraints, boosting fixed asset investment, and weakening corporate financial arbitrage motives. Further analysis demonstrates that the inhibitory effect of VAT rate reduction on corporate financialization is more pronounced for non-manufacturing companies, businesses reliant on the basic tax rate as their primary revenue source, companies with low intermediate input rates, and those with a strong ability to shift the tax burden. Additionally, debt financing costs play a crucial role in moderating the relationship between tax reduction policies and corporate financialization. The conclusions drawn from this study provide valuable empirical evidence that can contribute to the refinement of VAT reduction policies and the prevention and resolution of financialization at the micro-level.

## 1 Introduction

As China’s economy enters the "new normal," the investment environment for the industry is unfavorable, and the performance of real enterprises is unsatisfactory. Higher business risks, heavy tax burdens, and lower profit levels have seriously affected the healthy development of real enterprises. An increasing number of enterprises are changing their investment structure to achieve profit targets, injecting more capital into the virtual economy where costs and tax burdens are low and investment yields are higher and thereby deepening the financialization at the expense of industrial investment. Excessive financialization of enterprises will inhibit the development of the real economy, weaken their incentive and ability to innovate and exacerbate financial risks. The state has actively explored effective ways and taken many initiatives to curb the financialization of the real economy and to curb directing resources away from the real world.

As an integral component of active fiscal policy, tax policy serves as an effective instrument for countercyclical regulation, optimizing resource allocation, and consolidating economic development. In recent years, China has implemented various tax reduction policies aimed at supporting the growth of the real economy, fostering industrial investment, and curbing the financialization of the real economy. However, it is essential to examine whether these tax reduction policies can effectively achieve their objective of restraining the financialization of the real economy and redirecting corporate investment toward the industry. Furthermore, it is crucial to understand the underlying mechanisms through which these policies operate and whether their effects vary across different enterprises. Addressing these questions holds significant theoretical and practical implications, as it deepens our comprehension of tax reduction policies, provides sound recommendations for future policies, and effectively advances the financialization of the real economy.

This paper utilizes a sample of A-share listed enterprises from 2017 to 2020 to examine the impact of value-added tax (VAT) rate reductions in 2018 and 2019 on enterprises’ financialization. These rate reductions serve as significant representatives of the current tax reduction policy. The study employs a double difference model to empirically evaluate the influence of the tax reduction policy on enterprises’ financialization. In contrast to existing research, this paper contributes in the following ways: (1) Previous studies have predominantly focused on macroeconomic cycles and microenterprise characteristics when investigating factors affecting enterprises’ finances [1–4], with less attention given to the impact of enterprises’ tax burden. Our study explores the influence of VAT rate reduction on enterprises’ financialization from the perspective of tax reduction policy, thereby expanding the understanding of the factors affecting enterprises’ financialization.

(2) Our research contributes to the existing literature on the economic implications of tax policy. Previous studies have primarily examined the effects of tax reduction policy on corporate innovation and overall investment [5–7]. However, there is a scarcity of research that specifically investigates the impact of tax reduction on corporate financial assets and investment structure. In our study, we concentrate on assessing the influence of tax reduction policy on corporate financial assets and empirically examine the effect of reducing the VAT tax rate on enterprises. Additionally, we explore the pathway through which VAT reduction can curb financialization among enterprises. Our findings indicate that a decrease in the value-added tax rate restrains enterprises from transitioning towards virtual and speculative activities by alleviating corporate financing constraints, increasing fixed asset investment, and weakening the motivation for financial arbitrage.

(3) This paper contributes to the existing research on the relationship between tax reduction policies and corporate investment structure. Previous studies have predominantly focused on the correlation between taxation and productive corporate investment as well as overall investment [8, 9]. However, there is limited theoretical research that specifically investigates tax reduction as a support mechanism for exploring the impact of tax reduction on the financialization of nonfinancial corporate investment. In our study, we not only explore the impact of incremental tax reduction on enterprises’ financialization but also conduct further analysis on the effect of VAT rate reduction on financialization among enterprises with industry, different input and output structures, tax burden shifting ability, and debt financing costs. We delve into the variations in the impact of VAT rate reduction on enterprises’ financialization.

## 2 Literature review

### 2.1 Corporate financialization

From the perspective of investment structure, financialization of enterprises refers to nonfinancial enterprises investing more capital in the financial sector and allocating financial assets while reducing their investment in their main business [10]. From the perspective of investment structure, this refers to nonfinancial enterprises investing more in the financial sector, allocating less to their main business and gradually increasing the proportion of their financial assets from the perspective of revenue structure, this refers to nonfinancial enterprises increasing the proportion of their profits from financial sources, relying on financial investment income and neglecting the improvement of their main business.

From an investment structure perspective, the financialization of enterprises can be understood as nonfinancial companies allocating more capital to the financial sector and diverting financial assets away from their core business activities [11, 12]. Specifically, it entails nonfinancial enterprises increasing their investments in the financial sector, reducing their allocation to their primary business, and gradually raising the proportion of their financial assets [13, 14]. Furthermore, from a revenue structure standpoint, financialization refers to nonfinancial enterprises elevating the share of their profits generated from financial sources, relying heavily on financial investment income while neglecting the development of their core business [15, 16].

The existing literature predominantly focuses on examining the motives, influencing factors, and economic implications of enterprise financialization [17, 18]. The motives driving financialization can be categorized as "reservoir" and "investment substitutions." Financial assets, due to their high liquidity and the ability to be readily converted into cash, serve as a reservoir for enterprises to address future business uncertainties. When companies have surplus funds, they accumulate financial assets as a means of "storing water." Conversely, during periods of financial strain, they liquidate financial assets to alleviate liquidity pressures and generate funds for industrial investment [19, 20].

The motive of "investment substitution" entails firms seeking higher profits by allocating more capital to the financial sector when the returns on industrial investment are low, consequently diverting resources away from industrial investment [7]. Under this motive, the financialization of enterprises arises from the pursuit of greater profits and the excessive allocation of capital to financial assets for short-term gains. As a result, the investment and development of the core business of the enterprise are neglected, leading to insufficient investment in the real sector and severely impeding the development of the real economy [21, 22].

The phenomenon of financialization in enterprises is influenced by various factors, including macroeconomics [23], capital markets [24], and enterprise behaviors [25]. According to Deng, Zhang and Tang [26], firms may passively invest in financial assets as a survival strategy during economic shocks. However, a stable macroeconomic environment can effectively reduce this passive choice and mitigate the extent of corporate financialization. At the microlevel, factors such as executive incentives, managers’ personal traits, and employee share ownership plans play a significant role. Research by Du, Xie and Chen [27] suggests that executives’ financial arbitrage motivation increases under compensation incentives, leading to higher corporate investment in financial assets. Conversely, executives under equity incentives prioritize long-term corporate value growth over short-term returns, resulting in greater allocation of funds to areas such as research and development (R&D) rather than financial assets. Furthermore, Du and Zhou [28] find that executives with academic experience tend to prioritize rigor and stability, making them less likely to overinvest in financial assets and discouraging financialization. On the other hand, executives with military experience are more prone to overconfidence and adopt aggressive strategies, contributing to increased financialization within the firm [29]. Additionally, the implementation of employee share ownership plans can enhance employees’ sense of ownership, promote governance monitoring, and alleviate management agency problems. As a result, employee share ownership plans can effectively reduce excessive financialization and promote an optimal level of financialization.

The literature provides various perspectives on the economic consequences of enterprise financialization. For instance, observes that high returns on financial assets incentivize firms to allocate more capital to financial assets, thus "crowding out" industrial investment. Additionally, excessive financialization, characterized by a high proportion of investment in financial assets, can hinder fixed asset investment and innovation activities, consequently impairing the development of the core business of enterprises [19, 30]. Wang, Cao [31] find that financialization can enhance corporate profits. However, in the long term, it may undermine the incentive to innovate. Li [32] discovers that excessive investment in financial assets by listed companies increases the risk of share price fluctuations. Similarly, Liu and Ma [33] reach similar conclusions and affirm that corporate financialization, driven by excessive indebtedness, can trigger an increased risk of share price collapse.

### 2.2 Tax cuts and corporate behaviors

Tax reduction policies, as a crucial tool for macroeconomic regulation, have diverse impacts on the behavioral decisions of microenterprises. Existing studies examining the micro effects of tax reduction policies primarily focus on enterprise innovation and investment. Many scholars believe that tax cuts have a positive influence on corporate innovation activities. For instance, Gao, Zhang and Ni [34] conducted an empirical study and found that tax cuts promote corporate innovation by alleviating corporate financing constraints. Mao, Cao and Yang [35] examined the impact of the "camp reform" and observed that it optimized corporate cash flow by reducing corporate tax burdens, thereby facilitating corporate specialization, division of labor, and creating favorable conditions for corporate innovation. Employing counterfactual measures, Li [36] found that tax reduction policies contributed to innovation output by approximately 10% or more.

Furthermore, Li and He [37] investigated the effects of tax cuts on enterprises’ innovation output. They discovered that the implementation of tax cuts increased both intermediate goods output and finished goods output in enterprises’ innovation activities, demonstrating a long-term effect of such promotion.

The impact of corporate income tax cuts on corporate investment has been extensively studied in the literature. For instance, Auerbach and Hassett [38] analyzed micro firm data and found that the 1986 corporate income tax reform in the US had a significant effect on corporate investment activities. Chen and Fang [39] conducted a study on the accelerated depreciation policy for fixed assets and confirmed its positive effect on corporate investment. However, they observed that the implementation of the policy did not lead to improvements in investment efficiency.

On the other hand, there is relatively limited research on the effects of indirect tax policies such as value-added tax (VAT). Xu and Chen [40] investigated the VAT transformation reform and found that it had a substantial promoting effect on enterprise investment. They estimated that for every 1% point reduction in the effective VAT rate, enterprise investment increased by approximately 16%. Xiao [41], using a Difference-in-Differences (DID) model and stepwise regression method, found that VAT rate reduction significantly stimulated enterprise investment by reducing the burden of enterprise taxes.

### 2.3 Tax cuts and corporate financialization

The relationship between tax cuts and the financialization of enterprises has gained attention from scholars in recent years. Researchers generally argue that the corporate tax burden increases the investment in corporate financial assets and intensifies the level of corporate financialization [42]. Therefore, tax reduction can promote corporate investment in the real sector and effectively mitigate excessive financialization of enterprises [43]. For instance, Liu [44] conducted empirical research and found that a heavy tax burden reduces operating profits for enterprises, leading them to adjust their investment structure and allocate more funds to financial assets to achieve profit growth. It is worth noting that the government can effectively restrain the financialization of enterprises by reducing their tax burden and encouraging real sector investments. Li and Li [45] discovered that the implementation of tax reduction policies can limit the motives of "reservoir" and "investment substitution" for enterprises to invest in financial assets, thus curbing the deepening of financialization.

However, there are scholars who hold the opposite view, suggesting that implementing tax reduction policies can actually exacerbate the financialization of enterprises. For instance, Tu and Zou [46] argue that the cash-saving effect resulting from lower tax rates can increase the degree of financialization of enterprises instead of promoting physical investment.

### 2.4 Research gaps

Based on the review of the aforementioned papers, there are several gaps in the existing research, which motivate the need for further investigation: (1) Limited consideration of the corporate tax burden: Most studies on the financialization of enterprises have focused on the motives, influencing factors, and consequences of financialization, with less attention given to the impact of the corporate tax burden. The influence of tax policy on enterprises’ financialization needs to be explored, as it is an important factor that has been relatively overlooked. (2) Lack of emphasis on corporate investment structure: Existing research on the impact of corporate tax cuts has predominantly focused on corporate innovation and investment, while overlooking the specific analysis of corporate investment structure. This gap calls for further examination of how tax cuts affect the allocation of corporate investment and the composition of financial assets. (3) Effectiveness of tax policy in regulating corporate de-investment: The effectiveness of tax policy in addressing the issue of corporate de-investment and guiding enterprises to reduce their investment in financial assets to support the real economy remains a significant question. These aspects warrant careful consideration and investigation.

Considering the significance of value-added tax (VAT) reduction in the current tax reduction policy, this paper aims to address these gaps by focusing on the impact of tax cuts on enterprises’ financialization, specifically examining the VAT rate reduction policy. By empirically assessing the policy’s effect on enterprises’ financialization, this study aims to provide valuable insights and contribute to the existing literature in this area.

## 3 Theoretical analysis and research hypothesis

### 3.1 VAT reduction and enterprise financialization

Indeed, the tax burden faced by enterprises in China has been identified as a significant factor driving their increased financial investments and subsequent financialization [17, 44]. Enterprises often resort to increasing their financial asset investments in the virtual economy due to the perceived higher short-term profitability compared to their main business. This shift towards financial assets allows them to compensate for the lack of profitability in their core operations and pursue desired returns, leading to a trend of financialization. Consequently, the tax burden, being a fixed cost of business operations, exerts a dampening effect on enterprise industrial investment and becomes a pivotal factor pushing enterprises to prioritize virtual investments over real sector activities [42].

On the one hand, the high tax burden imposed on real enterprises leads to increased costs and diminished after-tax returns on investments, compelling enterprises to redirect their investment activities in pursuit of "income compensation" to maximize profits [47, 48]. Financial investment has emerged as an optimal avenue for enterprises to seek such income compensation, given its light asset allocation, short investment cycles, and high rate of return, it provides new avenues for income growth and aligns with their profit-seeking objectives [49, 50].

On the other hand, the financialization of enterprises is influenced by the incompatibility and mismatch between China’s current taxation system and the requirements of the new economic development mode. China’s existing tax system is primarily tailored to traditional physical transactions, and its slow adaptation to the changing landscape poses challenges in effectively regulating the growing number of emerging businesses and new transaction models. Consequently, tracking income within the virtual economy becomes arduous, and tax supervision becomes inadequate. This mismatch renders financial investment particularly attractive to real enterprises, effectively transforming it into a "tax haven." This allure is especially pronounced for enterprises burdened with a heavy tax load.

This demonstrates that the heavy tax burden compels enterprises to rely on financial channels to generate profits, leading to an increase in investments in the financial sector and a decline in investments in the industrial sector. Consequently, it further exacerbates the financialization of real enterprises. Therefore, tax reduction emerges as a viable strategy to stimulate the growth of the real economy and reverse the trend of enterprise financialization. Building upon the aforementioned analysis, we propose the following research hypothesis:

H1: A reduction in the VAT rate will have a substantial impact in curbing the financialization of enterprises.

### 3.2 VAT reduction, corporate finance constraints and corporate financialization

Corporate financialization’s "reservoir" motive refers to the increased investment in financial assets undertaken by firms as a precautionary measure against future liquidity shortages. A crucial precondition for this motive is the presence of cash flow constraints, where the corporate tax burden plays a significant role in exacerbating these constraints [45]. On one hand, a reduction in the VAT rate directly alleviates the corporate tax burden, leading to a decrease in current cash flow expenditures and an increase in operating cash flow. Consequently, this enhances the firm’s internal capital and mitigates internal financing constraints. On the other hand, the VAT rate cut can also influence external capital investment. An augmented operating cash flow improves the solvency of enterprises and their ability to distribute dividends. This policy demonstrates the government’s tax support for relevant industries, thereby enhancing external investors’ expectations regarding the prospects of enterprises [51]. Consequently, it facilitates the attraction of external capital, enhances the financing situation, and mitigates external financing constraints to a certain extent.

As internal and external financing constraints are alleviated, enterprises become less concerned about cash flow uncertainty. This reduces their inclination to "reservoir" their capital, leading to a decrease in investments in financial assets with high liquidity and an increased allocation of capital towards industrial investments. Consequently, this alleviates the level of corporate financialization. Based on the aforementioned analysis, we propose the following research hypothesis:

H2: A reduction in the VAT rate discourages corporate financialization by easing corporate financing constraints.

### 3.3 VAT reduction, corporate fixed asset investment and corporate financialization

In the presence of price elasticity of demand for goods, most firms are unable to fully transfer the VAT tax burden to the end consumers and instead share the burden with them. Through the reduction of the VAT rate, the government effectively allocates a portion of the tax revenue, which it would have otherwise received, to enterprises and consumers [41]. On one hand, the "concessions" provided to enterprises reduce cost pressures and enhance their operating cash flow. This, in turn, helps alleviate the financial constraints faced by enterprises in terms of their investment in fixed assets. As a result, it effectively encourages enterprises to increase their investments in fixed assets. On the other hand, the reduction in the tax rate leads to a decrease in the price level of goods [52]. The "concession" made to consumers stimulates consumer demand, thereby incentivizing enterprises to expand their operations and increase their supply. Consequently, this leads to an increase in investments in fixed assets. Under the influence of the VAT rate reduction, the greater the allocation of fixed assets by enterprises, the lower the relative proportion of financial assets, resulting in a reduced degree of corporate financialization.

Based on the aforementioned analysis, we propose the following research hypothesis:

H3: A reduction in the VAT rate discourages corporate financialization by increasing corporate investment in fixed assets.

### 3.4 VAT tax reduction, corporate financial arbitrage motive and corporate financialization

Some scholars have observed that given the unfavorable investment environment in the real sector and declining returns on investment, enterprises are increasingly driven by the motive of "investment substitution" to acquire financial assets, particularly in the context of China’s current problem of overcapacity in the real sector [53]. The underlying premise of the "investment substitution" motive is that enterprises seek to compensate for the lack of returns from real investments by generating profits through financial investments, which offer higher returns. This creates a financial arbitrage motive.

The reduction in the VAT rate has several effects in this regard. Firstly, it increases the cash flow of enterprises and reduces financing costs [54]. Secondly, it stimulates product sales, leading to increased profits and improved economic efficiency for enterprises. Additionally, the lowered VAT rate reduces the losses incurred by enterprises in cases where VAT is not deductible [55]. The combined impact of these factors enhances the overall profitability of enterprises’ operations, narrowing the gap between the returns from real and financial investments. Consequently, enterprises’ expectations and reliance on financial investments for profits are reduced, thereby weakening their incentive for financial arbitrage and effectively curbing their financialization.

Based on the analysis presented above, we propose the following research hypothesis:

H4: A reduction in the VAT rate discourages corporate financialization by weakening the incentive for corporate financial arbitrage.

## 4 Research design

### 4.1 Model construction

The reduction in the VAT rate is regarded as a natural exogenous experiment. Consequently, we adopt the double difference model (DID), a widely-used approach in policy evaluation, to examine the impact of the VAT rate reduction reform on the financialization of enterprises. This study focuses on enterprises operating in industries covered by the VAT rate reduction policy, comprising the experimental group. The control group consists of enterprises from other industries, allowing us to assess the reform’s effect by comparing the pre- and post-reform changes in the level of financialization. To test Hypothesis H1, we construct the following DID model (1), drawing inspiration from previous studies such as Xu, Pang and Zhang [56] and Xiao [41].


$${Fin}_{i,t}=\beta_{0}+\beta_{1}\times {Treat}_{i}\times {Policy}_{i,t}+\beta_{i}\times {Control}_{i,t}+{Comp}_{i}+{Time}_{t}+u_{i,t}$$ (1)

In the formula, the degree of financialization of the explained variable enterprise is denoted as Fin_i,t_. The key explanatory variable of interest in this paper is the interaction term *Treat*_*i*_×*Policy*_*i*,*t*_. The variable Treat represents the industry group, where a value of 1 indicates that the enterprise belongs to the industry affected by the VAT rate reduction, while a value of 0 indicates otherwise. The variable *Policy*_*i*,*t*_ represents the policy time and is set as 0 before the reform. Additional control variables in this model are represented as *Control*_*it*_. The individual fixed effect is denoted as *Comp*_*i*_, the time fixed effect is represented by *Time*_*t*_, and *u*_*i*,*t*_ denotes the residual term.

To examine the mediating mechanism of the VAT rate reduction policy on enterprises’ financialization, this study further tests Hypotheses H2, H3, and H4 using stepwise regression. Models (2) and (3) are constructed as follows.

$$X_{i,t}=\alpha_{0}+\alpha_{1}\times {Treat}_{i}\times {Policy}_{i,t}+\alpha_{i}\times {Control}_{i,t}+{Comp}_{i}+{Time}_{t}+u_{i,t}$$ (2)

$${Fin}_{i,t}=\gamma_{0}+\gamma_{1}\times {Treat}_{i}\times {Policy}_{i,t}+\gamma_{2}\times X_{i,t}+\gamma_{i}\times {Control}_{i,t}+{Comp}_{i}+{Time}_{t}+u_{i,t}$$ (3)

where *X*_*i*,*t*_ is the mediating variable (corporate finance constraints, fixed asset investment, financial arbitrage motive) to be tested.

1. If *α*_1_ in Eq (2) is significant, then *γ*_1_ in Eq (3) is not significant, and *γ*_2_ in Eq (3) is significant. The mediating variable is a major channel through which the VAT rate reduction policy affects corporate financialization. There is a full mediation effect.
2. If *α*_1_ in Eq (2) is significant, while *γ*_1_ and *γ*_2_ in Eq (3) are both significant, and the significance and coefficient of *γ*_1_ in Eq (3) are not significantly lower than those of *β*_1_ in Eq (1), then the mediating variable is an important channel through which the VAT rate reduction policy affects corporate financialization, and there is a partial intermediation effect.
3. If *α*_1_ in Eq (2) is significant and *γ*_2_ in Eq (3) is insignificant, then the mediating variable are not a channel for the effect of VAT rate reduction on corporate financialization.

### 4.2 Variable definitions and data descriptions

The specific definitions of the main variables in the model are provided below:

1. Explanatory variable: Degree of financialization of enterprises (Fin). This variable is measured based on a study by [42]. The financialization of nonfinancial enterprises is captured using a narrow definition of financial assets, which includes trading financial assets, loans or advances granted, held-to-maturity investments, and investment properties. Considering the growing prominence of the pan financial sector and the increasing divergence between the real economy and financial sector, this paper also incorporates long-term equity investments in enterprises as a manifestation of financialization. Therefore, the extent of financialization for an enterprise is determined by the proportion of the aforementioned financial assets in the enterprise’s total assets.
2. Explanatory variables: VAT rate reduction policy variables (Treat × Policy). Firstly, the industry dummy variable Treat is introduced. Industries that were subject to a 6% tax rate before and after the VAT rate reductions in 2018 and 2019 are considered the control group and assigned a value of 0. All other industries, excluding the control group, fall under the experimental group of the VAT rate reduction and are assigned a value of 1 for the industry dummy variable. Additionally, the VAT rate reduction policy timing variable Policy is introduced. It takes a value of 0 for the period from Q1 2017 to Q2 2018, representing the period before the tax rate reduction, and a value of 1 for the period after Q2 2019, representing the period after the two tax rate reductions. These variables serve as the core explanatory variables in this paper.
3. Mediators:
  ①SA, The degree of financing constraints faced by the firm is measured using the SA index, based on Hadlock and Pierce [57]. The calculation method is provided in Table 1.
  ②FAI, Based onXu, Pang and Zhang [56], FAI represents the change in net fixed assets from the beginning to the end of the period, reflecting the firm’s fixed asset investment. The calculation method is shown in Table 1.
  ③Driven: Driven represents the financial arbitrage motivation of enterprises. The calculation method is shown in Table 1. A higher index value indicates a stronger financial arbitrage motivation.
4. Control variables: The paper incorporates several control variables based on relevant literature (Peng, Han and Li 2018), as presented in Table 1.

**Table 1:** Names of variables and selection of indicators.

Variable type	Variable name	Variable symbols	Variable calculation formula
Explained variables	Level of corporate financialization	Fin	(Trading financial assets + loans or advances granted + held-to-maturity investments + investment properties + long-term equity investments)/total assets
Explanatory variables	Policy dummy variables	Treat×Policy	VAT rate reduction policy dummy variable
Mediators	Corporate finance constraints	SA	|-0.737Asset+0.043Asset2-0.04Age|
	Fixed asset investment	FAI	(Net fixed assets at the end of the period—Net fixed assets at the beginning of the period)/Total assets
	Financial arbitrage motive	Driven	(Investment income + fair value gain or loss + other comprehensive income—operating profit)/| Operating profit |
Control variables	Business size	Size	Natural logarithm of the total assets of the business
	Gearing ratio	Lev	Total liabilities/total assets
	Cash holding ratio	Cash	Cash and cash equivalents/total assets
	Management efficiency	ME	Administrative expenses/operating income
	Business Growth	TobinQ	The market value of business/total assets
	Return on total assets	ROA	Net profit/average total assets
	Individual fixed effects	Comp	Individual dummy variables
	Time fixed effects	Time	Time dummy variables

### 4.3 Sample selection

The data utilized in this paper were obtained from the CSMAR database and financial statements of Chinese listed companies. The sample consisted of nonfinancial, nonreal estate companies listed on the Shanghai and Shenzhen A-shares exchanges. The study focused on the period from the 1st quarter of 2017 to the 4th quarter of 2020.

To ensure data quality and consistency, the following data processing steps were conducted:

1. Exclusion of IPO and ST listed companies.
2. Removal of listed companies whose VAT tax rate belonged to the 13% industry before the abbreviated tax rate reform in 2017, specifically in the agriculture, forestry, fishery, and animal husbandry industry, as well as the electricity and heat industry. The remaining listed companies had VAT tax rates of 17%, 11%, or 6% based on their respective industries before the VAT reduction in 2018.
3. Elimination of observations with missing main variables.
4. Application of a 1% Winsor two-sided tailing process to continuous variables to mitigate the impact of extreme values.

After the data processing steps, a final dataset comprising 41,681 observations from 2,651 companies was obtained, covering the specified time period. Additionally, the industry classification used in this paper is based on the Chinese Securities Regulatory Commission’s 2012 Industry Classification Standard.

## 5 Analysis of empirical results

### 5.1 Analysis of basic regression results

The regression Model (1) was employed to estimate the relationship, and the results are presented in Table 2. The coefficients of the Treat×Policy variable exhibit significant negative effects, indicating that the VAT rate reduction policy has a significant impact on reducing the proportion of financial assets held by the enterprises affected by the policy. This reduction acts as a constraint on the financialization of these enterprises, thereby confirming Hypothesis H1. More specifically, the VAT rate reduction is associated with an approximate 2 percentage point decrease in the financialization degree of the sample firms.

**Table 2:** Base regression model results.

Variables	(1)	(2)	(3)	(4)
	Fin	Fin	Fin	Fin
Treat×Policy	-0.022*** (0.005)	-0.021*** (0.005)	-0.02*** (0.005)	-0.018*** (0.005)
Size			-0.002 (0.002)	-0.013*** (0.004)
Lev			-0.052*** (0.011)	-0.035*** (0.013)
Cash			-0.113*** (0.011)	-0.116*** (0.011)
ME			-0.003 (0.011)	-0.013 (0.011)
TobinQ			0.006*** (0.001)	0.006*** (0.001)
ROA			0.03 (0.018)	0.045** (0.019)
Constant term	0.041*** (0.001)	0.041*** (0.001)	0.11** (0.048)	0.35*** (0.091)
Time effect	Control	Control	Control	Control
Individual effects	No control	Control	No control	Control
Group R^2^	0.128	0.128	0.157	0.159

Note

*, ** and *** denote significance at the 10%, 5% and 1% significance levels, respectively, with robust standard errors in brackets.

The signs of the coefficients for the other control variables in the model generally align with theoretical expectations. However, for a more robust analysis that accounts for time and individual fixed effects, the regression results from Column (4) of Table 2 are selected as the basis for further examination.

The coefficient before the Size variable is significantly negative, aligning with theoretical expectations. It indicates that larger enterprises are less financialized compared to smaller enterprises. This can be attributed to the higher operating costs faced by smaller enterprises. In contrast, larger enterprises benefit from economies of scale, facing less cost pressure and operational challenges. Consequently, they focus more on their core business operations and allocate fewer resources to financial assets, thereby dampening the degree of financialization.

The coefficient of the Lev variable is significantly negative, implying a negative relationship between financialization and leverage ratio. Low financial leverage indicates lower financial risk and potential returns on investment. This encourages firms to invest more in financial assets to increase their returns while managing overall risk. Conversely, firms with high financial leverage often face capital constraints and have limited funds available for investment in financial assets.

The coefficient of the Cash variable is significantly negative, indicating a negative correlation between financialization and cash holdings. This can be explained by the substitution effect between financial asset holdings and cash holdings. When firms hold more cash, they have less incentive to invest in financial assets.

The positive coefficient of the TobinQ variable suggests that firms with high market value tend to invest more capital in financial areas with higher returns, aiming to achieve higher profitability. This contributes to their financialization.

The coefficient of the ROA variable is significantly positive, indicating a positive relationship between financialization and return on total assets. This reflects the surplus effect, where firms with higher profitability have more resources available for financialization activities [58].

The regression results for the ME variable are insignificant, suggesting that management efficiency does not have a significant impact on enterprises’ financialization.

### 5.2 Robustness tests

#### 5.2.1 Parallel trend test

To evaluate the effectiveness of policy implementation using a double difference model, it is crucial to ensure that the parallel trend assumption holds for both the experimental and control groups prior to policy implementation. To test the validity of the parallel trend assumption, the baseline regression model is augmented by including the interaction term between the dummy variable and the experimental group variable at each pre-policy implementation period. Subsequently, the regression is re-estimated. If the coefficient of the interaction term is not significantly different from zero before the introduction of the VAT rate reduction policy, it indicates that the parallel trend assumption is satisfied.

**Fig 1:**
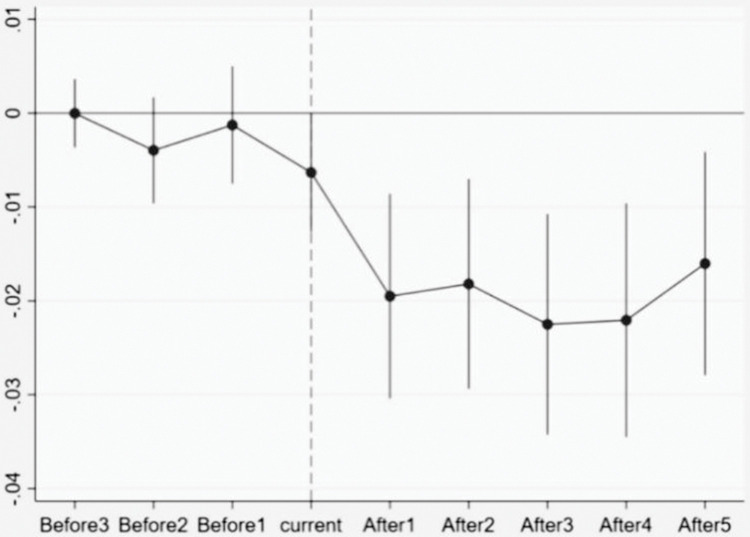
Parallel trend test.

Fig 1 presents the results of the parallel trend test. The horizontal axis represents the respective periods, while the vertical axis displays the regression coefficient of the interaction term. The periods are categorized as follows: "Current" refers to the period from the commencement of the first VAT rate reduction policy to the beginning of the second VAT rate reduction policy; "Before1," "Before2," and "Before3" represent periods 1, 2, and 3 before the first VAT rate reduction, respectively; and "After1," "After2," "After3," "After4," and "After5" correspond to periods 1, 2, 3, 4, and 5 after the second VAT rate reduction, respectively. The vertical lines in the figure indicate the corresponding 95% confidence intervals. It can be observed that the coefficients of the interaction terms are not significantly different from zero prior to the implementation of the VAT rate reduction policy, providing evidence in support of the parallel trend hypothesis. Furthermore, the coefficients of the interaction terms are significantly negative following the policy implementation, indicating a notable reduction in the financialization of enterprises after the two VAT rate reductions were implemented.

#### 5.2.2 Counterfactual test

To address potential confounding factors and ensure that the observed effects are indeed attributable to the VAT rate reduction policy studied in this paper, certain assumptions and methods are employed.

Firstly, it is acknowledged that factors other than the VAT rate reduction policy could potentially contribute to a reduction in the degree of financialization of enterprises. To mitigate this concern, the paper assumes that the VAT rate reduction policy is not substantially related to these other factors. Furthermore, to assess the robustness of the findings, the paper assumes that the VAT rate reduction policy is implemented six months or one year earlier than the actual implementation. By examining the significance of the regression results under these different assumptions, the paper aims to distinguish the specific impact of the policy from the influence of other factors. Moreover, in order to estimate the average treatment effect of the policy, the paper follows the approach suggested by Liu and Xu [59] and removes the sample data collected after the actual policy implementation. This counterfactual test helps to isolate the effect of the VAT rate reduction policy by comparing the outcomes with and without the policy.

The new estimates obtained from the Difference-in-Differences (DID) analysis, presented in Table 3, demonstrate that the coefficients of the interaction terms are no longer statistically significant. This suggests that the previous estimation results can be attributed to the effect of the VAT rate reduction policy itself, rather than hypothetical event shocks or random factors.

**Table 3:** Counterfactual tests.

	Timing of the implementation of the artificially changed VAT rate reduction			
	Six months in advance		One year ahead of schedule	
Variables	Fin	Fin	Fin	Fin
Treat×Policy	-0.0018 (0.0023)	-0.0018 (0.0022)	-0.0015 (0.0023)	-0.0022 (0.0022)
Control variables	No	Yes	No	Yes
Constant term	0.0408*** (0.0005)	0.3782*** (0.1153)	0.0408*** (0.0005)	0.3770*** (0.1155)
Time effect	Control	Control	Control	Control
Individual effects	Control	Control	Control	Control
Group R^2^	0.0134	0.0312	0.0134	0.0313

Note

*, ** and *** denote significance at the 10%, 5% and 1% significance levels, respectively, with robust standard errors in brackets.

#### 5.2.3 Replacement of explanatory variables

The classification of long-term equity investments in enterprise assets as financial assets or operating assets is subject to debate among scholars. While Peng, Han and Li [60] argue that holdings in financial institutions within long-term equity investments should be considered financial assets, Zhou and Xie [61] advocate for categorizing long-term equity investments as a whole as financial assets. On the other hand, most scholars contend that investments in subsidiaries, associates, and joint ventures serve operational purposes such as research and development, production, sales, and business expansion. As a result, they argue that long-term equity investments should be classified as operating assets.

To ensure the robustness of the regression results, this paper conducts an additional empirical analysis by reclassifying "long-term equity investment" as operating assets. The explanatory variables are replaced with Fin1 = (financial assets held for trading + loans or advances granted + held-to-maturity investments + investment properties)/total assets, and a new regression is performed. The results of this regression are presented in Table 4, demonstrating that they remain consistent with the hypothesis proposed in this paper. Specifically, the findings indicate that the reduction of the VAT rate significantly inhibits the financialization of enterprises.

**Table 4:** Replacement of explanatory variables.

Variables	(1)	(2)	(3)	(4)
	Fin1	Fin1	Fin1	Fin1
Treat×Policy	-0.015*** (0.004)	-0.014*** (0.004)	-0.013*** (0.004)	-0.011*** (0.004)
Control variables	No	NO	Yes	Yes
Constant term	0.013*** (0.001)	0.013*** (.001)	0.093*** (0.027)	0.186*** (0.060)
Time effect	Control	Control	Control	Control
Individual effects	No control	Control	No control	Control
Group R^2^	0.130	0.130	0.151	0.152

Note

*, ** and *** denote significance at the 10%, 5% and 1% significance levels, respectively, with robust standard errors in brackets.

### 5.3 Mediation effect test

#### 5.3.1 A test of the mediating effect of corporate financing constraints

In columns (1) and (2) of Table 5, the results of the mediation effect test for financing constraints are presented. In column (1), the dependent variable is financing constraints, and the regression coefficient of “Treat×Policy” is significantly -0.011. This indicates that the reduction in the value-added tax (VAT) rate effectively alleviates the financing constraints faced by enterprises. The first step of the mediation effect test confirms this finding. In column (2), the regression coefficient of “Treat×Policy”is significantly -0.019 at a 1% significance level. Therefore, it is evident that alleviating financing constraints serves as an important channel through which the VAT rate reduction policy inhibits the financialization of enterprises. This effect exhibits partial mediation. Thus, Hypothesis H2 is validated, supporting the notion that the reduction in the VAT rate effectively inhibits the shift of enterprises from real to virtual activities by alleviating their financing constraints.

**Table 5:** Mediation effect test results.

	(1)	(2)	(3)	(4)	(5)	(6)
Variables	SA	Fin	FAI	Fin	FAI	Fin
Treat×Policy	-0.011*** (0.002)	-0.019*** (0.005)	0.002*** (0.001)	-0.018*** (0.005)	-0.115*** (0.044)	-0.018*** (0.005)
SA		0.07* (0.037)				
FAI				-0.121*** (0.019)		
Driven						0.001*** (0.000)
Control variables	Yes	Yes	Yes	Yes	Yes	Yes
Constant term	3.581*** (0.128)	0.069 (0.157)	-0.139*** (0.017)	0.333*** (0.09)	-0.399 (0.718)	0.350*** (0.091)
Individual effects	Control	Control	Control	Control	Control	Control
Time effect	Control	Control	Control	Control	Control	Control
Group R^2^	0.794	0.144	0.065	0.160	0.097	0.159

Note

*, ** and *** denote significance at the 10%, 5% and 1% significance levels, respectively, with robust standard errors in brackets.

#### 5.3.2 A test of the mediating effect of corporate fixed asset investment

Columns (3) and (4) of Table 5 present the results of the mediation effect test for financing constraints. In column (3), the dependent variable is fixed asset investment of enterprises, and the regression coefficient of “Treat×Policy” is significantly 0.002. This implies that the reduction in the VAT rate significantly promotes fixed asset investment by enterprises. The first step of the mediation effect test supports this finding. In column (4), the regression coefficient of “Treat×Policy” is significantly -0.018 at a 1% significance level. Therefore, it is evident that promoting fixed asset investment serves as an important channel through which the VAT rate reduction policy inhibits the financialization of enterprises. This effect exhibits partial mediation. Thus, Hypothesis H3 is validated, indicating that the reduction in the VAT rate effectively inhibits the financialization of enterprises by increasing their fixed asset investment.

#### 5.3.3 A test of the mediating effect of corporate financial arbitrage motive

Columns (5) and (6) of Table 5 present the results of the mediation effect test for the financial arbitrage motive of enterprises. In column (5), the dependent variable is the financial arbitrage motive of enterprises, and the regression coefficient of “Treat×Policy” is significantly -0.115. This indicates that the reduction in the VAT rate significantly suppresses the financial arbitrage motive of enterprises. The first step of the mediation effect test confirms this finding. In column (6), the regression coefficient of “Treat×Policy” is significantly -0.018 at a 1% significance level. Therefore, it is evident that suppressing the financial arbitrage motive serves as an important channel through which the VAT rate reduction policy inhibits the financialization of enterprises. This effect exhibits partial mediation. Thus, Hypothesis H4 is validated, indicating that the reduction in the VAT rate effectively inhibits the financialization of enterprises by weakening their financial arbitrage motive.

### 5.4 Further analysis

#### 5.4.1 Industry heterogeneity test

The differential impact of VAT rate reduction on manufacturing and non-manufacturing enterprises is examined in Table 6. Non-manufacturing enterprises, being more labor-intensive and dependent on human resources, directly benefit from the reduction in labor costs and experience increased competitiveness and profitability. Additionally, the elasticity of demand for services is higher than that of manufacturing products, leading to increased consumer spending and sales growth for non-manufacturing firms. Consequently, the disincentive effect of VAT rate reduction on financialization is stronger for non-manufacturing enterprises.

**Table 6:** Industry heterogeneity test results.

	Manufacturing industry	Non-manufacturing
	Fin1	Fin1
Treat×Policy	-0.0003	-0.0261***
	(0.009)	(0.002)
Control variables	YES	YES
Constant term	0.1475***	0.4035***
	(0.033)	(0.047)
Time effect	Control	Control
Individual effects	Control	Control
r2	0.5881	0.7548

Note

*, ** and *** denote significance at the 10%, 5% and 1% significance levels, respectively, with robust standard errors in brackets.

The results in Table 6 confirm this finding. The regression coefficient of the interaction term "Treat×Policy" for non-manufacturing enterprises is significantly negative, passing the 1% significance level. In contrast, for manufacturing enterprises, the regression coefficient of "Treat×Policy" is not significant, indicating that the VAT rate reduction has a less pronounced restraining effect on financialization in the manufacturing sector. Thus, it can be concluded that the VAT rate reduction has a stronger inhibitory effect on the financialization of non-manufacturing enterprises.

#### 5.4.2 Heterogeneity based on input and output structure test

The impact of the VAT rate reduction policy can vary based on the differences in enterprises’ input and output structures. To examine the heterogeneous effects caused by these differences, the study refers to Tu [62] and distinguishes the output and input items of enterprises.

The difference in the tax rate applied to a firm’s main business is used to reflect variations in output items. Enterprises primarily engaged in basic rate businesses are covered by the policy and experience a reduction in output tax. Conversely, enterprises mainly involved in businesses with lower tax rates undergo a smaller or unchanged reduction in tax rates. As a result, their sales tax reduction is significantly lower compared to enterprises primarily engaged in businesses subject to the basic tax rate.

The disparity in the intermediate input rate of enterprises reflects differences in input structure. A higher intermediate input rate indicates a higher rate of taxation on inputs. In such cases, the reduction in input tax due to the tax rate reduction may be greater or even exceed the reduction in output tax. Consequently, the actual VAT liability may increase instead of decrease, undermining the intended purpose of tax reduction and failing to produce the desired effect.

The test results presented in Table 7 reveal important insights. Firstly, in the group of firms subject to the basic tax rate, the regression coefficient before Treat×Policy is negative and statistically significant at the 1% level. This finding indicates that the VAT rate reduction policy has a significant inhibitory effect on the financialization of these firms. Conversely, in the group of firms subject to the low tax rate, the regression coefficient of Treat×Policy is not statistically significant, suggesting that the reform does not have a significant impact on the financialization of these firms. Consequently, it can be concluded that the VAT rate reduction has a stronger inhibitory effect on the financialization of firms primarily engaged in basic tax rate businesses.

**Table 7:** Heterogeneity analysis of input and output structure.

	Basic tax rate applies of businesses	Low tax rate applies of businesses	Low intermediate input rates of businesses	High intermediate input rates of businesses
	Fin1	Fin1	Fin1	Fin1
Treat×Policy	-0.017*** (0.005)	**-0.013** (0.020)	-0.024*** (0.007)	-0.010 (0.006)
Control variables	Yes	Yes	Yes	Yes
Constant term	0.399*** (0.096)	0.275** (0.108)	0.405*** (0.156)	0.334*** (0.102)
Time effect	Control	Control	Control	Control
Individual effects	Control	Control	Control	Control
R^2^	0.171	0.151	0.192	0.124

Note

*, ** and *** denote significance at the 10%, 5% and 1% significance levels, respectively, with robust standard errors in brackets.

Secondly, when considering the intermediate input rate, the regression coefficient before Treat×Policy is negative and statistically significant at the 1% level in the group of firms with a low intermediate input rate. This finding suggests that the VAT rate reduction policy significantly curbs the financialization of these firms. However, in the group of firms with a high intermediate input rate, the regression coefficient of Treat×Policy is not statistically significant. This indicates that the reform does not have a significant impact on the financialization of these firms. Thus, it can be inferred that the VAT rate reduction has a stronger inhibitory effect on the financialization of firms with a low intermediate input rate.

#### 5.4.3 Heterogeneity based on the ability to pass the tax burden test

The VAT system imposes tax burdens on enterprises throughout the supply chain, and the distribution of the final tax burden at each stage is crucial. The ability of enterprises to pass on this tax burden determines who benefits from and to what extent they benefit from the VAT rate reduction, thus leading to different policy effects. This ability to pass on the tax burden is closely related to the bargaining power of enterprises. Enterprises with strong bargaining power possess the ability to negotiate favorable terms, allowing them to lower purchase prices and increase sale prices to shift the tax burden. Consequently, these enterprises can enjoy a larger share of the VAT reduction dividend and adjust their investment decisions accordingly. Thus, it is hypothesized that the reduction in VAT rates will have a stronger disincentive effect on the financialization of enterprises with greater tax-shifting power.

In this study, the bargaining power of upstream and downstream enterprises is considered, and the pass-through tax ability of enterprises is measured using the concept of supply chain concentration. Supply chain concentration is calculated as the average of the proportion of purchases from the top 5 suppliers and the proportion of sales to the top 5 customers. Regression analyses are conducted separately for two groups: the strong tax pass-through group (enterprises with tax pass-through ability exceeding the overall median level) and the weak tax pass-through group.

The results, as presented in Table 8, demonstrate that the regression coefficient before Treat×Policy is significantly negative in the group with stronger tax pass-through ability. Conversely, in the group with weaker tax pass-through ability, the regression coefficient before Treat×Policy is not statistically significant, indicating that the reform does not have a significant impact on the financialization of these enterprises. These findings suggest that the VAT rate reduction has a stronger dampening effect on the financialization of enterprises with stronger tax pass-through ability.

**Table 8:** Heterogeneity analysis of tax burden shifting capacity.

	Enterprises with strong tax pass-through capacity	Enterprises with weak tax pass-through capacity
	Fin1	Fin1
Treat×Policy	-0.025***(0.008)	-0.004(0.006)
Control variables	Yes	Yes
Constant term	0.548***(0.172)	0.158*(0.096)
Time effect	Control	Control
Individual effects	Control	Control
Group R^2^	0.176	0.151

Note

*, ** and *** denote significance at the 10%, 5% and 1% significance levels, respectively, with robust standard errors in brackets.

#### 5.4.4 Moderating effect of debt financing cost

For companies facing higher debt financing costs, the act of taking on debt implies higher thresholds, more restrictions, and increased pressure to repay the debt. These factors inevitably impose greater constraints on physical investments such as fixed assets. Conversely, lower costs of debt financing alleviate financing constraints, leading to increased productivity, efficiency, and a greater willingness to invest in fixed assets. This, in turn, reduces investments in financial assets and strengthens the disincentive for financialization. Consequently, the effect of the VAT rate reduction policy on the disincentive for corporate financialization is likely to be stronger when considering the heterogeneity of debt financing costs.

To examine the moderating effect of debt financing costs on the inhibitory effect of the value-added tax (VAT) rate reduction policy on corporate financialization, the following model is constructed:

$${Fin}_{i,t}=\beta_{0}+\beta_{1}\times {Treat}_{i}\times {Policy}_{i,t}+{\beta_{2}\times {Treat}_{i}\times {Policy}_{i,t}\times {R\_int}_{i,t}+\beta_{3}\times {R\_int}_{i,t}+\beta }_{i}\times X_{i,t}+{Comp}_{i}+{Time}_{t}+u_{i,t}$$ (4)

In the equation, *R*_*int*_*i*,*t*_ represents the measure of heterogeneity factor, which is the debt interest rate. It is calculated as the ratio of current financial expenses to total liabilities at the end of the period.

We examine the empirical regression results reported in Table 9. Of particular interest is the regression coefficient of the interaction term *Treat*_*i*_×*Policy*_*i*,*t*_×*R*_*int*_*i*,*t*_, which captures the moderating effect of debt financing costs. The results reveal that this interaction term has a significantly negative coefficient at the 1% level, providing evidence for the moderating effect of debt financing costs. Specifically, the VAT rate reduction policy has a stronger inhibitory effect on corporate financialization in firms with higher debt financing costs.

**Table 9:** Results of the test for the moderating effect of debt financing cost.

Variables	Model 8	Model 8
	Fin	Fin
Treat×Policy	-0.017***(0.005)	-0.015***(0.005)
Treat×Policy×R_int	-1.022***(0.144)	-1.012***(0.142)
R_int	0.770***(0.105)	0.737***(0.098)
Control variables	No	Yes
Constant term	0.04***(0.001)	0.346***(0.091)
Individual effects	Control	Control
Time effect	Control	Control
Group R^2^	0.135	0.165

Note

*, ** and *** denote significance at the 10%, 5% and 1% significance levels, respectively, with robust standard errors in brackets.

## 6 Conclusion

The excessive financialization of enterprises poses a risk to the development of the real economy. To promote the growth of the real economy, the government has implemented various policies, including tax reduction measures such as VAT rate reduction. This study focuses on the VAT rate reduction implemented in 2018 and 2019 in China, which serves as a representative tax reduction policy. By employing a double difference model and analyzing a sample of A-share listed entities from 2017 to 2020, the study reveals several key findings.

Firstly, the reduction in the VAT rate has a significant inhibitory effect on enterprises’ financialization, this is consistent with the findings of [63]. However, while their study used data from Chinese industrial enterprises (2000–2012), our research is based on Chinese A-share listed companies (2017–2020). This not only broadens the scope of the study’s subjects but also updates the data for recent years. Through this, we have verified the restraining effect of value-added tax reform on corporate financialization. This conclusion is not only applicable to Chinese enterprises but can also offer insights for tax policy reforms in other countries.

Secondly, the impact of China’s Value-Added Tax (VAT) reform on corporate financialization is particularly pronounced among non-manufacturing firms, firms applying the basic tax rate, firms with a low intermediate input rate, and firms with a strong ability to shift tax burdens. Conversely, there is no significant effect observed among non-manufacturing firms, firms applying the low tax rate, firms with a high intermediate input rate, and firms with a weak ability to shift tax burdens. This research conclusion further enriches the literature on the effects of Value-Added Tax (VAT) reform on corporate behavior. The studies conducted by [64, 65] have verified the impact of VAT reform on various aspects of corporate behavior, including investment behavior, production efficiency, and business trade. In contrast, our study goes a step further by extending the investigation to explore the influence of this system on the financialization behavior of different types of enterprises.

Additionally, the study demonstrates that the cost of debt financing plays a positive moderating role in the relationship between the VAT rate reduction and the financialization of firms. Specifically, higher debt financing costs strengthen the dampening effect of the VAT rate reduction on corporate financialization. Finally, the study identifies three channels through which the VAT rate reduction inhibits enterprises’ financialization. These channels include the alleviation of financing constraints, increased investment in fixed assets, and a decrease in financial arbitrage incentives. Our study complements the findings of [66]. They indicated that Value-Added Tax (VAT) reform significantly enhanced firms’ external financing capabilities. In addition, our research further reveals that VAT reform reduces corporate financialization by alleviating firms’ financing constraints.

## 7 Empirical implications

First, previous studies have demonstrated the effectiveness of tax reduction policies in suppressing enterprises’ financialization. The empirical findings of this article further reveal that in addition to conventional direct tax policies, indirect tax policies like VAT play a significant role in promoting the integration of financialization and the real economy. Hence, it is crucial to recognize the potential of indirect tax policies, such as VAT, in driving the financialization of the real economy.

Second, considering that VAT reduction policies exhibit diverse effects on enterprises’ financialization, local governments should tailor their policies based on four key factors: industry characteristics, input and output structure, tax burden transfer capability, and debt financing costs. By exploring complementary tax and fee reduction policies, they can effectively facilitate the development of the real economy.

Third, given that VAT reduction effectively curbs corporate financialization by alleviating financing constraints, stimulating investment in fixed assets, and reducing incentives for financial arbitrage, it is recommended to not only promote VAT tax reduction and fee reduction policies but also enhance support for the real economy. This can be achieved by attracting external investors and optimizing the investment environment of the virtual economy, thus fostering a symbiotic relationship between the virtual and real economies.

## 8 Limitations and future research

Since this study is based on empirical research focusing on China’s Value-Added Tax (VAT) reform policy, although the research findings offer certain policy implications for other countries, it remains unclear whether VAT reforms in different countries yield comparable effects. Therefore, it is necessary for future research to expand the sample size further and explore the diversity in the impact of various countries’ tax reforms on corporate financial dynamics. Moreover, this study exclusively examines the impact of the VAT policy, a tax reduction measure, on corporate financialization. In the future, it is essential to extend the investigation to examine the effects of other tax reduction and fee reduction policies (such as individual income tax and social insurance contributions) on corporate financialization.
